# Developing and validating a risk prediction model for acute care based on frailty syndromes

**DOI:** 10.1136/bmjopen-2015-008457

**Published:** 2015-10-21

**Authors:** J Soong, A J Poots, S Scott, K Donald, D Bell

**Affiliations:** 1 NIHR CLAHRC Northwest London, Imperial College London, Chelsea and Westminster Campus, London, UK; 2 Royal College of Physicians, London, UK; 3 Oliver Wyman, London, UK

**Keywords:** Frailty Syndromes, Risk Prediction, Acute, Outcomes, Model

## Abstract

**Objectives:**

Population ageing may result in increased comorbidity, functional dependence and poor quality of life. Mechanisms and pathophysiology underlying frailty have not been fully elucidated, thus absolute consensus on an operational definition for frailty is lacking. Frailty scores in the acute medical care setting have poor predictive power for clinically relevant outcomes. We explore the utility of frailty syndromes (as recommended by national guidelines) as a risk prediction model for the elderly in the acute care setting.

**Setting:**

English Secondary Care emergency admissions to National Health Service (NHS) acute providers.

**Participants:**

There were N=2 099 252 patients over 65 years with emergency admission to NHS acute providers from 01/01/2012 to 31/12/2012 included in the analysis.

**Primary and secondary outcome measures:**

Outcomes investigated include inpatient mortality, 30-day emergency readmission and institutionalisation. We used pseudorandom numbers to split patients into train (60%) and test (40%). Receiver operator characteristic (ROC) curves and ordering the patients by deciles of predicted risk was used to assess model performance. Using English Hospital Episode Statistics (HES) data, we built multivariable logistic regression models with independent variables based on frailty syndromes (10th revision International Statistical Classification of Diseases, Injuries and Causes of Death (ICD-10) coding), demographics and previous hospital utilisation. Patients included were those >65 years with emergency admission to acute provider in England (2012).

**Results:**

Frailty syndrome models exhibited ROC scores of 0.624–0.659 for inpatient mortality, 0.63–0.654 for institutionalisation and 0.57–0.63 for 30-day emergency readmission.

**Conclusions:**

Frailty syndromes are a valid predictor of outcomes relevant to acute care. The models predictive power is in keeping with other scores in the literature, but is a simple, clinically relevant and potentially more acceptable measurement for use in the acute care setting. Predictive powers of the score are not sufficient for clinical use.

**Strengths and limitations of this study**

- It is a simple clinical model that has moderate predictive powers outcomes relevant to acute medical care. It has reduced data requirements compared with existing frailty models trialled in the acute care setting with predictive powers evenly spread over three outcomes.
- It is a model designed to be that could be applied at point of access to acute care, does not rely on self-reported data and was derived from whole population data that are routinely collected.
- This study adds to emerging knowledge surrounding the secondary use of administrative data. It provides a novel methodology to best utilise routinely collected data in a systematic and robust manner that minimises limitations and optimises data quality and reliability.
- Hospital Episode Statistics (HES) is retrospectively coded, thus reflects the patient's condition at discharge from hospital.
- Diagnostic coding accuracy in HES has been challenged.

## Introduction

In the majority of countries, the population is living to a greater age. This change in population demographics is not necessarily associated with failing health as individual variation exists. A recent survey indicates that the majority of those over 80 years are satisfied or very satisfied with their health.^1^ For some, however, this is associated with an increase in comorbidity^2^ and functional dependence,^3^ with a consequent higher health and social care cost. A large component of this increased need is reflected in hospital demand both for elective and non-elective care. Patients over the age of 65 constitute two-thirds of admissions, 40% of all hospital bed days and 65% of National Health Service (NHS) spend in acute care.^4^ Within this population, there is group of patients that most clinicians and the public would regard or recognise as frail and at higher risk of adverse outcomes.

Much research has taken place in understanding the pathophysiology and mechanisms underlying frailty;^5^ ^6^ however, assessing frailty reliably remains problematic and is a research priority.^7–13^ This is compounded at present by the absence of consensus on an operational definition of frailty.^14–16^ Two broad approaches to measuring frailty have been described; a specific biophysical phenotype(unintentional weight loss, exhaustion, weakness, slowness and low physical activity)^17^ and an index of accumulated deficit model.^18^ These models have the benefit of reproducibility, and predict important health outcomes such as mortality, self-reported health and functional dependency.^19^ Though overlap exists between these models,^20^ to date, published scores based on these operational definitions demonstrate only poor to moderate predictive powers for adverse outcomes within the acute medical care setting.^9^ Developing a reliable and clinically acceptable method to quantify frailty that links to outcomes would help in clinical practice as well as provide a method for longitudinal population analysis.

Within elderly care, there are a number of syndromes that are commonly recognised in older person, including ‘Giants of geriatrics’^21^ or geriatric syndromes.^5^ These are common clinical presentations of multifactorial ill-defined processes recognised in older persons. They include cognitive impairment, pressure ulcers, mobility problems, falls and incontinence. Conceptually, they represent a final common pathway of concentric, non-linear processes formed by the interaction between aetiological and physiological mechanisms, as yet not fully elucidated.^5^ When complex systems fail, high-order systems tend to break down first.^22^ This potentially makes frailty syndromes a robust marker for this vulnerable patient cohort. In the acute care setting, they are associated with increased functional dependence and length of hospital stay.^23^ Current national guidelines for the care of the older person in acute care recommend using frailty syndromes as a possible methodology to assess for frailty.^11^ ^12^

This study explores the hypothesis that frailty syndromes are a valid measure of adverse health outcomes in older persons within the acute care population in England using routinely available secondary care data based on Hospital Episode Statistics (HES).^24^ We aim to develop and validate a model of frailty based on these syndromes as the first steps of developing a sensitive clinically relevant assessment tool to be used at point of access of acute care. We aim to evaluate its predictive power for clinical outcomes relevant to acute medical care. For construct validity,^25^ we explore its association with the Charlson comorbidity score.^26^

## Methods

### Data source

HES is an administrative data set collected for the secondary care setting that has high levels of data completeness and rigorous data cleaning processes, ensuring high data quality. Each record in HES corresponds to a finished consultant episode, during which a patient is under the care of an individual consultant. These episodes were aggregated into hospital spells covering the entirety of a patient's length of stay in a hospital using established methodology.^27^

HES contains 20 fields per record for diagnoses codes that are defined in the 10th revision of the International Statistical Classification of Diseases, Injuries and Causes of Death (ICD-10). We systematically explored all 20 diagnostic fields within HES for ICD-10 diagnostic codes to group together to form frailty syndromes (see online supplementary appendix 1). To explore the effect of coding shifts over time within HES (thereby potentially affecting coding reliability), annual trend profiles for the grouped ICD-10 diagnostic codes were plotted from January 2005 to March 2013 (see online supplementary appendix 2). As a result of this analysis, data from the years 2010–2012 were selected for the final model, and we merged ICD-10 diagnostic codes for dementia, delirium and senility to form a unified frailty syndrome (cognitive impairment).

Emergency admissions were defined as those for which the method of admission was recorded as ‘Emergency’, either via accident and emergency services, a general practitioner, a Bed Bureau, a consultant outpatient clinic or other means (HES Column header: *admimeth*=21, 22, 23, 24, 28).

The final risk prediction model included all spells for patients over 65 years with emergency admission to English NHS acute providers from 01/01/2012 to 31/12/2012 (N=2 099 252).

### Model input and output variables

Table 1 describes predictor variables for study, including patient demographics, frailty syndromes and previous service use. Table 2 describes outcome variables under investigation, including inpatient mortality, 30-day emergency readmission and increase functional dependence at discharge (measured as a change in discharge destination to an institution providing more social and functional support when compared with admission source). In the UK, residential homes are care homes that provide accommodation, meals and some personal care. Nursing homes are residential care homes, but additionally have registered nurses that provide care for more complex needs. English care homes can be privately owned, third sector, local authority or NHS owned. In England, cost for local authority part 3 residential accommodation is charged to the resident.

**Table 1:** Predictor inputs for frailty risk prediction model (independent variables)

Name	Time span	Description	Comments
Age	Current spell	The startage field from HES	
Sex	Current spell	The sex field from HES	
Admission source	Current spell	The admiSorc field from HES	
Charlson (historic)	24-month historic average	Calculated per spell, using all diagnoses from all episodes and then averaged. Excludes the current spell	
Charlson (current)	Current spell	Calculated using diagnoses in positions 2–20 from all episodes in the spell	
Anxiety and depression	24-month historic binary indicator	A binary flag indicating whether a relevant diagnosis has been received during any inpatient spell in the past 24 months	Senility, dementia and delirium merged to form the cognitive impairment indicator because of changes in coding over time
Cognitive impairment			
Dependence			
Falls and fracture			
Incontinence			
Mobility problems			
Pressure ulcers			
Number of emergency admissions	12-month historic count	The number of emergency admission spells in the previous 12 months, excluding the current spell	Normalised
Days since last emergency admission	24-month historic	The number of days since the patient's last discharge from an emergency admission	Normalised. Default value used when the patient has not had an emergency admission in the previous 24 months

HES, Hospital Episode Statistics.

**Table 2:** Predictor outputs of frailty risk prediction model (dependant variables)

Name	Time span	Description	Comments
Inpatient mortality	Current spell	Indicates if the discharge destination was death	
30-day emergency readmission	30 days from discharge	Indicates if the patient had an emergency admission within 30 days of discharge from the current spell	
Increase in functional dependence	Current spell	Binary outcome indicates if the patient's discharge destination was associated with a higher level of functional dependence than the admission source	See functional dependence tiers below

Tier	Values in tier
1	The usual place of residence, including no fixed abode Temporary place of residence when usually resident elsewhere, for example, hotels and residential educational establishments
2	Local authority part 3 residential accommodation: where care is provided Non-NHS (other than Local Authority) run residential care home
3	NHS run nursing home, residential care home or group home Non-NHS (other than Local Authority) run nursing home
4	NHS other hospital provider: ward for general patients or the younger physically disabled or A&E department Non-NHS run hospital
5	Non-NHS (other than Local Authority) run hospice

A&E, accident and emergency; NHS, National Health Service.

The model consisted of both historical and within-spell variables. Historical variables included data up to 24 months prior to admission spell in 2012, while within-spell variables were only measured during the patients’ admission spell in 2012. Historical diagnostic codes were chosen over in-spell ones when coding for frailty syndromes as this more accurately described a risk prediction model at the point of access to acute care. Charlson comorbidity scores were calculated in HES using previously described methodology,^28^ using weightings originally described by Charlson.^26^

Spells ending with inpatient mortality were excluded when predicting institutionalisation or readmission within 30 days. Spells where the admission source or discharge destination could not be allocated a tier were also excluded when calculating functional dependence (approximately <1% of spells not ending in mortality).

### Model development and testing

Pseudorandom numbers split patients into train (60%) and test (40%) groups. We then split spells into train (1 259 185 spells) and test (840 067 spells) sets based on the groupings (to ensure no patient appears in both train and test sets). Multicollinearity between predictor variables was investigated by variance inflation factor (VIF), where VIF scores of over 3 were taken to denote unacceptable collinearity. Scikit-learn^29^ implementation of logistic regression with l2 regularisation was used to create the risk prediction model. The model coefficients selected in the train set were then used to score all samples in the test set. Finally, receiver operator characteristic (ROC) curves and area under the curve (AUC) scores^30^ were generated based on the predicted probabilities within the test set scores. Hosmer-Lemeshow^31^ tests with scipy implementation of Pearson's χ^2^ test were performed for goodness-of-fit. Ordering the patients by deciles of predicted risk allows a visual representation of the models discrimination.

## Results

### Mortality

**Figure 1:**
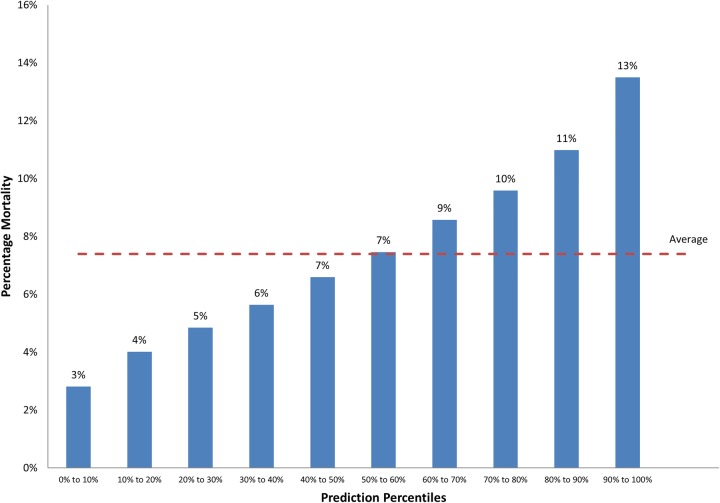
Percentage mortality by prediction ranking for the frailty syndromes and admission history model.

None of the models predictor variables (patient demographics, frailty syndromes, previous service use) demonstrated unacceptable collinearity (1.1–2.8) (table 3). Table 4 describes the predictive power of various frailty syndrome models for within-spell inpatient mortality (range of AUCs 0.624–0.659). The frailty syndromes and admission history model demonstrates moderate discriminatory power, with the top 10% of patients identified at highest risk of inpatient mortality having a mortality rate (13%) nearly twice the average population (7%; figure 1). The addition of Charlson comorbidity score did not significantly improve the predictive power of the model (AUC=0.641). However, in-spell Charlson and frailty syndrome models described slightly improved predictive power over historical models (tables 4 and 5).

**Table 3:** Variance inflation factor scores for predictor variables

Variance inflation factor scores	
Age	2.6
Sex	1.8
Historic Charlson	1.1
Anxiety and depression	1.7
Cognitive impairment	1.1
Dependence	1.6
Fall	1.1
Incontinence	1.2
Mobility	1.1
Pressure ulcers	1.8

**Table 4:** Frailty syndrome models to predict within spell in-patient mortality

Model	ORs		AUC
Historical frailty syndromes model	Age	1.05	0.624
	Sex	1.30	
	Anxiety and depression	0.94	
	Cognitive impairment	1.21	
	Functional dependence	1.11	
	Falls and fracture	0.94	
	Incontinence	1.06	
	Mobility problems	1.08	
	Pressure ulcers	1.29	
In-spell frailty syndromes model	Age	1.05	0.659
	Sex	1.20	
	Anxiety and depression	0.93	
	Cognitive impairment	1.40	
	Functional dependence	0.64	
	Falls and fracture	0.65	
	Incontinence	1.34	
	Mobility problems	1.16	
	Pressure ulcers	4.04	
			
Historical frailty syndromes and Charlson comorbidity scores	Age	1.05	0.641
	Sex	1.09	
	Charlson	1.20	
	Anxiety and depression	0.98	
	Cognitive impairment	1.01	
	Functional dependence	1.02	
	Falls and fracture	0.97	
	Incontinence	1.01	
	Mobility problems	1.01	
	Pressure ulcers	1.05	
Historical frailty syndromes and admission history (final model)	Age	1.05	0.632
	Sex	1.21	
	Anxiety and depression	0.95	
	Cognitive impairment	1.05	
	Functional dependence	1.04	
	Falls and fracture	0.90	
	Incontinence	1.02	
	Mobility problems	1.02	
	Pressure ulcers	1.11	
	Number of emergencyadmissions (12 months)	0.97	
	Days since last emergency admission	0.79	

AUC,area under the curve.

**Table 5:** Charlson comorbidity models to predict within spell in-patient mortality

Model	ORs		AUC
Historic Charlson	Age	1.05	0.639
	Sex	1.31	
	Charlson	1.20	
In-spell Charlson	Age	1.05	0.681
	Sex	1.02	
	Charlson	1.29	

AUC, area under the curve.

### Discharge to a higher level of support

**Figure 2:**
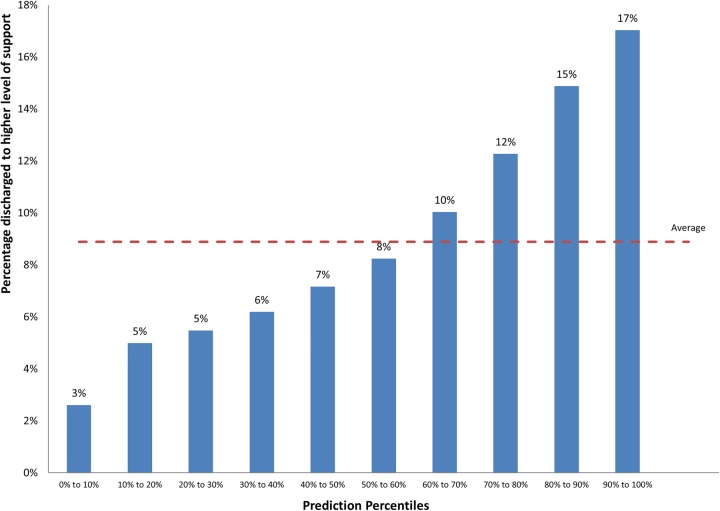
Percentage discharged to a higher level of functional dependence (institutionalisation) by prediction ranking for the frailty syndromes and admission source model.

Table 6 describes the predictive power of frailty syndrome models to predict discharge to a higher level of support (institutionalisation; range of AUCs 0.63–0.654). The frailty syndromes and admission source model demonstrated moderate discriminatory power, with the top 10% of patients identified at highest risk of being discharged to a higher level of support (17%) at nearly twice the average population (9%; figure 2). Historic Charlson comorbidity scores (taking into account age and gender) exhibited AUCs of 0.617.

**Table 6:** Frailty syndrome models to predict discharge with a higher level of support (institutionalisation)

Model	ORs		AUC
Historic frailty syndromes and admission history	Age	1.04	0.634
	Sex	0.94	
	Anxiety and depression	0.98	
	Cognitive impairment	1.36	
	Functional dependence	1.20	
	Falls and fracture	1.15	
	Incontinence	1.09	
	Mobility problems	1.12	
	Pressure ulcers	1.20	
	Number of emergency admissions (past 12 months)	0.82	
	Days since last emergency admission	0.98	
Historic frailty syndromes and admission source	Age	1.04	0.654
	Sex	0.94	
	Admission source (×5)	0.42–2.60	
	Anxiety and depression	0.94	
	Cognitive impairment	1.36	
	Functional dependence	1.17	
	Falls and fracture	1.14	
	Incontinence	1.08	
	Mobility problems	1.16	
	Pressure ulcers	1.17	
			
Historic frailty syndromes	Age	1.05	0.63
	Sex	0.95	
	Anxiety and depression	1.02	
	Cognitive impairment	1.24	
	Functional dependence	1.05	
	Falls and fracture	1.18	
	Incontinence	1.04	
	Mobility problems	1.09	
	Pressure ulcers	1.04	

AUC, area under the curve.

### Thirty-day emergency readmission

**Figure 3:**
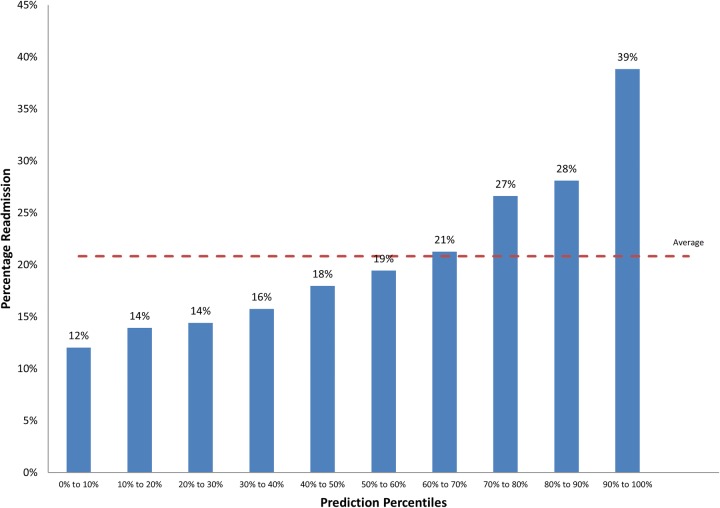
Percentage with emergency readmission within 30 days by prediction ranking for the frailty syndromes and admission history model.

Table 7 describes the predictive power of the frailty models to predict emergency readmission within 30 days (range of AUCs 0.57–0.63). The frailty syndromes and admission history model demonstrated moderate discriminatory power, with the top 10% of patients identified at highest risk of emergency readmission within 30 days (39%) at nearly twice the average population (21%; figure 3). Historic Charlson comorbidity scores (taking into account age and gender) exhibited AUCs of 0.591.

**Table 7:** Frailty syndrome models to predict emergency readmission within 30 days

Model	ORs		AUC
Historic frailty syndromes	Age	1.00	0.574
	Sex	1.20	
	Anxiety and depression	1.55	
	Cognitive impairment	1.24	
	Functional dependence	1.11	
	Falls and fracture	1.25	
	Incontinence	1.11	
	Mobility	1.35	
	Pressure ulcers	1.15	
Historic frailty syndromes and admission history	Age	1.00	0.630
	Sex	1.12	
	Anxiety and depression	1.08	
	Cognitive impairment	1.05	
	Functional dependence	1.02	
	Falls and fracture	1.03	
	Incontinence	1.02	
	Mobility	1.06	
	Pressure ulcers	1.02	
	Number of emergency admissions (last 12 m)	1.47	
	Days since last emergency admission	0.67	

AUC, area under the curve.

## Discussion

Risk stratification of older persons who require acute care is complex and challenging. Reliable recognition of frailty is a research and clinical priority for acute hospital care^7–13^ to help inform routine clinical decision-making and plan appropriate care. To date, there is no routinely available and reliable clinical score for use within the acute care setting. This study explores the use of internationally recognised frailty syndromes coded within HES data to potentially aid more reliable frailty recognition within the hospital setting. HES data can reliably provide data related to mortality and high resource need (eg, occupied bed days or readmission). We have constructed a surrogate marker of functional dependency (ie, institutionalisation) using available HES fields. The ideal frailty assessment for acute care needs to be comprehensively multidimensional to avoid missing aspects of patient care that may contribute to further decline or harm. It needs to predict outcomes that are relevant to the patient, carers and to acute care providers. To be fit for purpose, it should be optimised for clinical usability: that is, simple, reliable, does not fully rely on self-reported or carer-reported data and possess high sensitivity if functioning as a screening tool. Ideally, there should be the ability to personalise the assessment and ‘threshold’ set to patient preference and previous level of functioning. It should be able to provide a method to measure frailty over the course of an episode of acute illness and over a patient's life as opposed to single isolated static measures. Ultimately, it should be able to highlight areas for intervention to prevent, reverse or minimise further decline.

Studies exploring the predictive power of frailty scores for outcomes relevant to the UK acute medical care setting (table 8) include prospective observational cohort studies^8^ ^9^ ^32^ and secondary analysis of routinely collected large data sets, both clinical^33^ and administrative.^28^ ^34^ Our model performs uniformly across the clinical outcomes and is comparable in predictive power to frailty scores in the same setting. None of the models have predictive powers suitable for clinical risk prediction at the patient's bedside (AUC >0.80). The exception to this is a single study in the Acute Medical Unit (AMU) setting in rural Ireland,^35^ which reported AUCs of >0.8 for 30-day mortality and functional decline, but the results of this secondary analysis of a clinical database was not reproduced in prospective observational study at a large teaching centre in the UK.^10^

**Table 8:** Summary of the predictive power of frailty scores in acute care

Model/scores AUCs	Mortality		Readmission		Functional dependence	
	Inpatient	90-day	30-day	90-day	Institutionalisation	≤2 points Barthel ADL
Charlson score 2012 (historic)	0.64		0.59		0.62	
CHS model		0.61		0.52	0.57	0.55
SOF model		0.59		0.53	0.44	0.56
Avila-Funes		0.68		0.55	0.50	0.59
Rothman		0.67		0.53	0.45	0.59
Frailty Index		0.69		0.57	0.55	0.57
ISAR		0.62		0.60	0.65	0.60
PARR30			0.70			
RIGAMA	0.78		0.55		0.50	
Frailty syndrome models						
Frailty syndromes and admission source					**0.65**	
Frailty syndromes	**0.62**		**0.57**		**0.63**	
Frailty syndromes and admission history	**0.63**		**0.63**		**0.63**	

ADL, Activities of Daily Living; AUC, area under the curve; ISAR, Identifying Seniors at Risk; PARR, Patient At Risk of Readmission 30-Day; RIGAMA, Risk Index for Geriatric Acute Medical Admission.

Bold typeface indicates AUC for Frailty Syndrome Models.

Our model has notable strengths. It is a simple clinical model that has moderate predictive power outcomes relevant to acute medical care. It has less data requirements compared with the Frailty Index (36 input variables),^9^ Patient At Risk of Readmission 30-Day (PARR30; up to 18 input variables),^34^ Risk Index for Geriatric Acute Medical Admissions (RIGAMAs; 30 input variables)^33^ and Charlson comorbidity score (17 input variables).^28^ Importantly, in comparison to other scores, its predictive power appears to be evenly spread over the three outcomes and does not rely on self-reported data (eg, Identifying Seniors at Risk (ISAR) score).^36^ It is a model designed to be that could be applied at point of access to acute care. It was derived from whole population data that is routinely collected, with applicability at population and patient level. This study adds to emerging knowledge surrounding the secondary use of administrative data. It provides a novel methodology to best utilise routinely collected data in a systematic and robust manner that minimises limitations and optimises data quality and reliability.

Existing frailty scores in the acute care setting have very different input variables (thus likely do not measure the same thing). Optimal outcome variable selection is also yet unclear. For example, our model and most existing frailty scores do not take into account illness severity or disease acuity. We postulate that the addition of variables included in the National Early Warning Score (NEWS)^37^ may improve discrimination of frailty models. RIGAMAs^33^ notable predictive powers for inpatient mortality may reflect discrimination for acute critical illness given input variables that largely record physiological and metabolic derangement, including prognostic biomarkers (eg, troponin). However, it may be that the optimal outcome variable for frailty in acute care is 30-day or 90-day mortality.

Studies of frailty scores in the emergency department (ED) setting display similar predictive powers for a wide range of outcomes: *HK-ISAR* >65 years discharged from ED AUC 0.59–0.62 for composite outcome of institutionalisation, reattendance or death^38^; *ISAR* score >65 years admitted to hospital via ED AUC 0.549–0.584,^39^ AUC 0.66 for depressive symptoms, AUC 0.61–0.68 for frequent ED visits, AUC 0.66–0.68 for frequent hospitalisation, AUC of 0.71 for frequent use of community services,^40^ high acute care utilisation AUC 0.68;^41^ Triage Risk Screening Tool (TRST) score AUC 0.626–0.640 and Variable Indicative of Placement risk (VIP) score AUC 0.588–0.654 for functional decline >65 years admitted to hospital via ED;^39^ Score Hospitalier d'Evaluation du Risque de Perte d'Autonomie (SHERPA) for >70 years admitted via ED AUC 0.73 for functional decline at 3 months;^42^ Hospital Admission Risk Profile (HARP) >70 years admitted to hospital AUC 0.65 for functional decline;^43^

Studies of frailty scores in the hospital ward setting report slightly better predictive powers, but these scores might reflect a subselected (and therefore possibly more frail), and in most instances, older patient population: >70 years admitted to geriatric unit by clinical judgement for composite outcome of mortality OR admission to residential care facility OR transfer from low to high care within residential facility at discharge frailty index of accumulated deficits (FI-CD) AUC 0.735, *Katz* AUC 0.704, Cardiovascular Health Study (CHS) AUC 0.675, Study of Osteoporotic Fractures (SOF) AUC 0.679, Fatigue, Resistance Ambulation, Illness, Loss of Weight (FRAIL) AUC 0.638, ten-domain frailty index based on Comprehensive Geriatric Assessment (FI-CGA-10) AUC 0.617*, Gait* AUC 0.643, *SHERPA* AUC 0.697, Multi-dimensional prognostic index (MPI) AUC 0.617 *HARP* AUC 0.639 Charlson's co-morbidity index (CCI) AUC 0.579;^44^ >50 years admitted to Intensive Care Unit (ICU) Clinical Frailty Scale (CFS) ORs for in-hospital mortality(1.81), adverse events(1.54), 1-year mortality(1.82), low quality of life score(1.98) and functional dependence(2.25);^45^ *FI* for patients admitted with hip fracture AUC 0.82 for failure to return home at 30 days;^46^ >65 years admitted to hospital MPI AUC 0.76, FI-SOF AUC 0.68, FI-CD AUC 0.73, FI-CGA AUC 0.72 for all-cause mortality at 1 month;^47^ >80 years admitted to hospital for at least 48 h via ED AUC 0.81 for functional decline at 2 months;^48^ >70 years admitted to acute geriatric ward CHS OR for mortality at 6 months *CHS* (4.68), *SOF*(1.97); >75 years admitted to acute care hospital, for every 1% increase in *FI* is associated with a 5% increase in risk of death.^49^

We noted a phenomenon of improved predictive power reflected with in-spell models compared with historic models for both Charlson comorbidity scores and frailty syndromes. There may be two causes. First, HES data are coded at discharge, not admission. Diagnostic coding in HES may improve throughout the patients in-hospital stay with in-spell coding methodology adding an extra admission as a window for this to happen. Second, there may be ‘leak’ from the primary diagnostic coding position as these complex patients will likely have several reasons for emergency admission to hospital. Interestingly, taking into account comorbidity (by way of Charlson comorbidity score) did not significantly improve predictive power. VIF scores suggest only mild collinearity between the Charlson comorbidity score and frailty syndromes, suggesting mild overlap between the variables.

All our models displayed significance at p<0.05 for the Hosmer-Lemeshow tests for goodness-of-fit test. Similar findings have been described by others who have produced models on HES specifically^28^ as the test is recognised to detect unimportant differences within large data sets.^50^ Ordering the patients by deciles of predicted risk allows a visual representation of the models discrimination.

## Limitations

Though HES is a large data set with high information standards, it has limitations. It is retrospectively coded, thus reflects the patient's condition at discharge from hospital. To counter this, the model inputs data from historic spells to more accurately reflect a risk prediction tool at point of entry to care. Diagnostic coding accuracy in HES has been challenged. Plotting annual trend profiles of the data allowed us to choose a suitable temporal range to develop the model, as well as account for any change in coding practices over time. Even so, the administrative data set may not accurately reflect the actual clinical situation. Coding inconsistencies will limit the models predictive powers and accuracy. Prospective testing on a clinical data set is a necessary next step. Though a rich data set, HES does not contain variables previously identified as being predictive of frailty (eg, polypharmacy or weakness). This risks excluding potentially relevant variables from the model.

HES does not record specific clinical measures of functional dependency (eg, Barthel Index). The creation of a five-tier discharge institution levels represents a pragmatic approach to create an outcome that reflects increase in care need (within HES) as a proxy measure for increase in functional dependency. The premise of comparing discharge institution to admission source within HES as a surrogate for functional dependency is possibly flawed. Cohort and epidemiological studies suggest that there is significant overlap of functional dependency between residents of residential and nursing homes. Additionally, thresholds for transfer into and out of homes in the residential care setting are highly context and health system dependant. For instance, there is marked variation in the manner that criteria for NHS long-term funding are applied between geographical settings. However, the model adds new knowledge surrounding methodologies to utilise routinely collected data for answering clinically meaningful questions.

## Conclusion

Frailty syndromes are a valid predictor of outcomes relevant to acute care. We provide a frailty score developed from routinely collected administrative data, and this study adds further understanding and utility for the secondary use of these data. The models predictive power is in keeping with other scores in the literature, but is a simple, clinically relevant and potentially more acceptable measurement for use in the acute care setting. Predictive powers of the score are not sufficient for clinical use, though HES coding quality in HES may be responsible. Prospective testing in a clinical data set and the addition of other variables known to predict frailty may improve predictive power. Frailty is an important dimension in risk stratification of older persons requiring acute care.
